# Environmental-social-economic footprints of consumption and trade in the Asia-Pacific region

**DOI:** 10.1038/s41467-020-18338-3

**Published:** 2020-09-08

**Authors:** Lan Yang, Yutao Wang, Ranran Wang, Jiří Jaromír Klemeš, Cecília Maria Villas Bôas de Almeida, Mingzhou Jin, Xinzhu Zheng, Yuanbo Qiao

**Affiliations:** 1 Fudan Tyndall Center and Shanghai Key Laboratory of Atmospheric Particle Pollution and Prevention (LAP3), Department of Environmental Science & Engineering, Fudan University, 200438 Shanghai, China; 2 Institute of Eco-Chongming (SIEC), No.3663 Northern Zhongshan Road, 200062 Shanghai, China; 3 Faculty of Engineering Technology at University of Twente, Enschede, 7500 AE The Netherlands; 4 Sustainable Process Integration Laboratory (SPIL), NETME Centre, Faculty of Mechanical Engineering, Brno University of Technology (VUT Brno), Technická 2896/2, 61669 Brno, Czech Republic; 5 Universidade Paulista, UNIP R. Dr. Bacelar, 1.212, 4th Floor, 04026-002 Sao Paulo, Brazil; 6 Industrial and Systems Engineering Department, Institute for a Secure and Sustainable Environment, The University of Tennessee at Knoxville, 525D John D. Tickle Engineering Building, Knoxville, TN 37996 USA; 7 School of Economics and Management, China University of Petroleum-Beijing, 102249 Changping, Beijing China; 8 Institute for Studies in County Development, Shandong University, 266200 Qingdao, China

**Keywords:** Climate-change policy, Environmental economics, Environmental impact, Socioeconomic scenarios

## Abstract

Asia-Pacific (APAC) has been the world’s most dynamic emerging area of economic development and trade in recent decades. Here, we reveal the significant and imbalanced environmental and socio-economic effects of the region’s growths during 1995–2015. Owing to the intra-regional trade of goods and services, APAC economies grew increasingly interdependent in each other’s water and energy use, greenhouse gas (GHG) and PM_2.5_ emissions, and labor and economic productivity, while the environmental and economic disparity widened within the region. Furthermore, our results highlight APAC’s significant role in globalization. By 2015, APAC was engaged in 50–71% of the virtual flows of water, energy, GHG, PM_2.5_, labor, and value added embodied in international trade. While the region’s final demand and trade grew less resource- and emissions-intensive, predominantly led by China’s transformations, APAC still lags behind global averages after two decades. More joint efforts of APAC economies and attention to sustainable transformation are needed.

## Abstract (web summary)

The environmental and socio-economic implications of the growth in welfare and trade in Asia-Pacific (APAC) remain unclear. Here the authors show that over the past two decades (1995–2015), owing to intraregional trade, the APAC economies have grown increasingly interdependent in natural resource use, air emissions, and labor and economic productivity.

## Introduction

The growth of global supply chains has profoundly altered how raw materials are obtained, commodities are produced, traded and consumed, and hence the geographic distributions of income gains and environmental pressures^1,2^. As the most dynamic emerging area, the Asia-Pacific (APAC) region occupies a crucial position in the global supply chain, being a prominent supplier of raw materials, intermediate goods, and labor to the rest of the world^3,4^. APAC’s contribution to global exports increased from 28.7% in 2000 to 36.1% in 2018. APAC’s intraregional merchandise exports accounted for 57.7% of total exports in 2018, up from 50.8% in 2000^5^. The Belt and Road Initiative (BRI)^6^ and the Regional Comprehensive Economic Partnership (RCEP), a proposed free-trade agreement in the APAC region and the world’s largest trade deal when operational^7,8^, demonstrate the considerable efforts that have been made to strengthen the bonds of economic growth through trade within the region.

Despite the promising economic welfare, wide-ranging environmental, societal, and economic sustainability challenges loom in the APAC region. For instance, the groundwater irrigation is a prime source of income for India, Bangladesh, China, Pakistan, and Nepal and jointly accounts for approximately 50% of the global groundwater use^9^. However, the overexploitation of groundwater, especially in arid areas of those countries, has far exceeded the sustainable limit, threatening existing food and energy productions^10,11^. An earlier study highlighted that, during 1970−2005, APAC’s rapid economic growth and decreasing resource efficiency had been a major driver for overshooting the resource utilization limits globally^12^. It was also estimated that, international export caused nearly 208,500 mortalities related to air pollution^13^. Moreover, the densely populated APAC is one of the world’s most disaster-prone regions (e.g., hurricanes, droughts, flooding, and tsunamis). Nearly half of the world’s natural disasters in 2018 occurred in the region and, on average, 42 million people were affected, and $ 675 billion economic losses were caused per year^14^. Income inequality is also profound^15^. Despite leading the world in poverty alleviation, APAC has not been able to narrow income inequalities during rapid economic growth, with the Gini coefficient rising from 0.34 in 1990–1994 to 0.38 in 2010–2014^16^. Closing the environmental, social, and economic development gaps are critical for the region to achieve the 2030 Agenda for Sustainable Development and its Sustainable Development Goals (SDGs)^17^.

Our knowledge regarding the influences of regional economic welfare on global resource use and environmental emissions has grown substantially in recent years. A key development is the consumption-based environmental footprint accounting that links regional consumption to natural resource exploitation and the environmental impacts both within and outside of the region. Existing footprint studies vary in focal indicators: most of them focused on a single indicator (e.g., blue water^18^, energy^19^, carbon^20^, PM_2.5_^21^, land^22^, labor^23^, and material extraction^24,25^), and a few examined multiple-indicators consistently (e.g., carbon-land-water^26^ and carbon-land-water-material^27^). They also differ in temporal coverage, with most studies being based on a 1-year snapshot^28,29^, and a few investigated the time trends^24,30^. Large economies, such as the United States^31^, China^32^, and the European Union^26^, have been the focus of some studies. However, knowledge for the APAC region remains limited to scattered information within global information of a single indicator^33–37^ (see a brief literature review in Supplementary Table 1). For example, they show that carbon-emissions-intensive products manufactured in developing countries, such as China and India, were imported and eventually consumed by developed countries, such as the United States and Western European countries^38^. The employment footprint estimates indicate many Asian countries are servants to support the lifestyle of master countries^3^. The APAC countries’ environmental-social-economic bonds joined by the growing intraregional trade and the environmental-social-economic implications of APAC’s ever-increasing trade with the rest of the world remain poorly understood. Moreover, the socio-economic implications, which correspond to the other two dimensions of sustainability (aside from environment), have been largely neglected in the existing footprint research, leaving a crucial knowledge gap for assessing and improving regional sustainability.

Here, we analyze the patterns and trends of how the growths in economic wellbeing and trade activities of APAC during 1995–2015 affected the environmental-social-economic footprints of the APAC region. Our analysis is based on EXIOBASE 3.6, providing consistent time series of Multi-Regional Input Output (MRIO) tables with rich product detail for countries/regions covering the entire world^39^. In the database, APAC is represented by seven individual countries/regions (Australia, China, India, Indonesia, Japan, South Korea, and Taiwan, China), accounting for 80−90% of APAC’s population and GDP in 2015^40^. Other relatively small economies are aggregated as one region, RoAP (Rest of Asia-Pacific). Crucially, we select six indicators to measure the utilization, dependence, and pressures on natural resources (blue water consumption and energy use), local and global environmental threats (PM_2.5_ and greenhouse gas (GHG) emissions), and socio-economic effects (employment and economic value added). The six indicators form a footprint family that corresponds to the environmental-social-economic dimensions of sustainability, and SDGs (e.g., SDG 6, 7, 10, and 13). For over half the 17 SDGs, APAC’s progress is stagnant or heading in the wrong direction, hence urgent action is needed to accelerate its progress^41^. An analysis of the environmental and socio-economic implications of the region’s consumption and trade can provide a better understanding of APAC’s transformation from internal bonds and external relationships. Such understanding is also relevant for implementing the sustainability prospect of the BRI. Our results indicate that the intraregional trade has exacerbated the environmental and economic disparity within APAC over the past two decades, while the environmental externalities have been primarily outsourced to the lower-income economies, the higher-income economies have dominated and accounted for an increasing share of the economic gains of intra-APAC trade. Globally, APAC’s engagement in the virtual flows of water, energy, GHG, PM_2.5_, labor, and value-added embodied in trade reached unprecedented levels, i.e., 50% or higher. Although APAC’ final demand and trade has grown less resource- and emissions-intensive, primarily led by China, APAC still lags behind global averages by 2015. There is an urgent need to alleviate the imbalance of APAC’s intraregional development, and avoid ecological damages in already vulnerable areas.

## Results

### Heterogeneous and affluence-influenced footprints of APAC

**Fig. 1:**
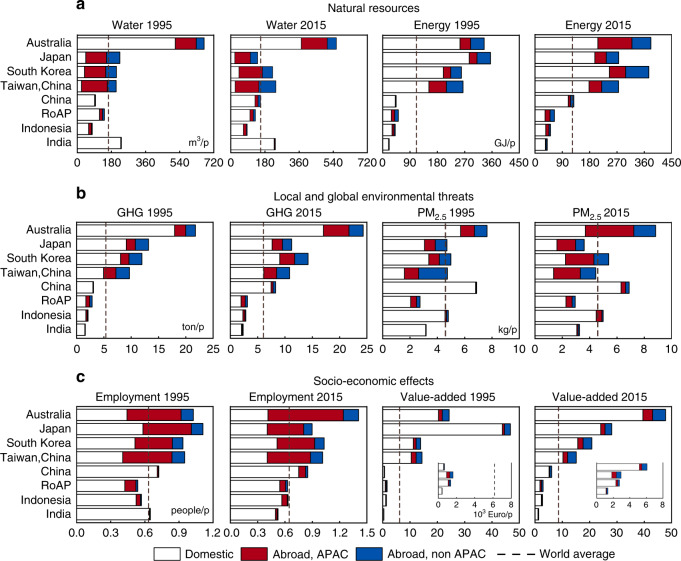
The environmental-social-economic footprints of APAC countries/regions in 1995 and 2015. The six footprint indicators fall into three categories and are presented in **a** natural resource, **b** local and global environmental threats, **c** socio-economic effects. The eight countries/regions are aligned on the *y*-axis following a descending order by average income per capita in 2015. Footprints are further broken down to the impacts occurred within the country/region, abroad in other APAC countries/regions, and abroad in non-APAC countries/regions. In each subplot, the dashed line indicates the world average level. GHG (CO_2_, N_2_O, and CH_4_) are measured in CO_2_ equivalent based on the 100-year Global Warming Potential (GWP100). Note, in the plots for value added, we use the insets to show the footprint compositions of the last four countries/region. China refers to Chinese mainland, and we call Taiwan, China as Taiwan for short in the rest.

The amount of natural resource extractions, environmental emissions, and socio-economic influences associated with satisfying the final demand of average person vary significantly among the eight APAC countries/regions (Fig. 1). The variations correlate with differences in affluence levels (Supplementary Table 2), which is consistent with the findings of previous footprint research focusing on GHG emissions^4^, and blue water consumption^42^ across countries worldwide. While the footprints of lower-income countries (e.g., India, Indonesia, RoAP, and China) have also increased with poverty alleviation, yet, most of them are still below the global averages by 2015. In addition, based on the varying environmental footprints (i.e., blue water, energy, and GHG) of the region’s six countries assessed annually from 1995–2015, we find some evidence for the Environmental Kuznets Curve (EKC) hypothesis, i.e., environmental pollution first rises and then falls as economic development proceeds (Supplementary Fig. 1 and Supplementary Fig. 2). Previously, few studies have addressed the socio-economic implications driven by final demand, such as the labor inputs required (employment) and revenues generated (value added)^1^. Here we find that a country/region’s employment and value-added footprints are both positively correlated with its affluence level.

Affluence levels also affected the geo-compositions of the footprint indicators in the APAC region. Richer APAC economies showed high and increasing reliance on outsourcing blue water consumption, PM_2.5_ emissions, and labor abroad, especially within the APAC region. Specifically, 58, 56, and 52% of the blue water footprints of Japan, South Korea and Taiwan were traced to water consumed in other APAC economies in 2015 (Fig. 1), primarily through the intra-APAC trade of agricultural products, such as sugar cane/sugar beet and paddy rice (Supplementary Fig. 3). In contrast, the final demand of middle- to low-income APAC economies has been largely satisfied by domestic natural and labor resources over the two decades. Moreover, during the same period, the PM_2.5_ footprint of high-income APAC economies (Australia, Japan, South Korea, and Taiwan) that occurred in other APAC economies increased from ~17 to 40%. Yet, for the less wealthy countries (e.g., China, India, and Indonesia), 75−99% of PM_2.5_ footprints were indigenous, a considerable fraction of which are attributed to direct household emissions (Supplementary Fig. 3). The consumption of traditional fossil fuels and biomass (e.g., fuelwood and agriculture wastes) for home cooking and heating, and the open-air combustion of biomass, especially in rural areas of India and China has been considered a significant source of PM_2.5_ emissions^43–45^.

Among all the indicators, we find the value-added footprints of the APAC economies, i.e., the contributions of their final demand to global economic growth (domestic + intra-APAC + non-APAC) experienced the most significant increases. Such increases are especially significant for the middle- and low-income APAC economies. For example, China’s per capita value-added footprint increased by eight times during 1995−2015, followed by India (217%), Indonesia (113%), and RoAP (82%). The rates are much higher than those experienced by the high-income economies (28% on average).

The APAC economies have grown more interdependently linked during the past two decades. Depending on the footprint indicators, the foreign footprint shares traced to APAC countries (Abroad, APAC in Fig. 1) increased by 4–27% while the domestic shares decreased by 3–33%. Given that the compositional changes within the aggregated RoAP cannot be estimated, the variation of RoAP is not included in the ranges. The increased intra-APAC interdependencies are primarily attributable to the strengthened linkages among the six major APAC countries. In contrast, India’s environmental-social-economic footprints remained predominantly domestic (90–99% in 1995 and 82–97% in 2015). Previously, researchers highlighted that resource constraints have already become a bottleneck for India’s social and economic development, such as fresh water scarcity^46^ and energy deficiency^47^. Air quality deterioration caused by PM_2.5_ emissions has also made India under severe health burden^48^. Our results here confirm that India remains highly dependent on local resource use and labor-intensive production activities to sustain its socio-economic growth. Engaging in international trades has the potential of reducing the depletion of local scare resources (e.g., blue water and energy) through import while adding employment and added-value through exporting goods produced with abundant labor resources and low environmental impacts locally^1,30^. The trade-environment relationship is primarily rooted in the economic principle of competitive advantages among countries for international trade. Therefore, adopting the strategy of reducing trade barriers rationally, increasing the openness to the outside world to actively promote international economic cooperation may be one solution to alleviating the pressure of domestic resource depletion (i.e., water and energy) and environmental damages (i.e., carbon and air pollutions) during India’s economic growth.

### Footprint outsourcing and disparity through intratrade

**Fig. 2:**
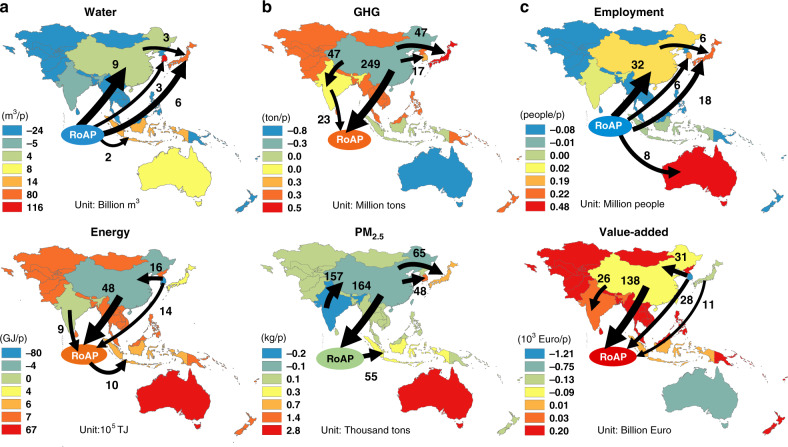
Net environmental-social-economic virtual flows of the intra-APAC trade in 2015. The top five net flows are shown for each footprint indicator, and fall into three panels **a** natural resource, **b** local and global environmental threats, **c** socio-economic effects. The width of the arrows in each panel represents the magnitude of the net flow within the APAC region. The background colors indicate the specific net footprint (import-export) per capita of each region/country. The negative net footprint indicates net displacements (of resource use, emissions, labors, and economic value added) to other APAC countries/regions. Note, the background color and flows of Taiwan, China could not be shown on the map.

The linkages and imbalances among the APAC countries/regions, through the virtual flows of environmental-social-economic resources embedded in intra-APAC trade, are highlighted in Fig. 2 (2015) and Supplementary Fig. 4 (1995). The intra-APAC trade patterns observed over the past two decades confirm that developed economies (Japan, Australia, South Korea, and Taiwan) import natural resources and labor from less-developed regions where resources and labors are cheaper (China, India, and RoAP). In 2015, for the 37 billion m3 of net bilateral virtual water trade in the region, nearly 80% was associated with the exports from RoAP and India, while ~50% was driven by the final demand of Japan and South Korea, mainly embedded in a range of water-intensive agricultural crops and products. Yet, as mentioned above, India has already been threatened by serious water crises with low availabilities of safe drinking water^49^. For the 15 EJ net bilateral energy flow embedded in the 2015 intra-APAC trade, China was the main net supplier to the rest of the region, contributing 43%, followed by South Korea (27%), which possesses strong petrochemical and steel industries. China’s net energy outflows were predominately embedded in energy-intensive products (e.g., coal power, steel, and gasoline), 73% of which ultimately served RoAP’s final demand. Of the 488 Tg net bilateral flows of GHG emissions, 76% was supplied by China, while the final demand of RoAP contributed more than half of it (286 Tg), and the rest is attributed to the final demand of four higher-income economies (Australia, Japan, South Korea, and Taiwan, 123 Tg in total). 81% of the 677 thousand tons of net bilateral PM_2.5_ flows occurred in China and India. And 38% of the net flows were driven by the final demand of four higher-income economies. The social and economic effects of the intra-APAC trade are more nuanced than those related to natural resources and emissions. The Intra-APAC trade resulted in a net bilateral outpouring of 99 million people, i.e., labor resources, in 2015. RoAP was the largest net labor supplier, contributing 77%, while Australia, Japan, South Korea, and Taiwan had net inflows of labor to satisfy their final demands, equivalent to employing 11.4, 27.6, 9.3, and 5.6 million people from the rest of APAC, respectively. In terms of net flows of value added, the intraregion pattern appears significantly different and almost opposite from those of other indicators. By net outpouring value added to other APAC economies, China turned from the second trade surplus country in 1995 to the largest one within APAC in 2015, followed by the developed economies. Yet, on the per capita basis, South Korea, Australia, Japan and Taiwan achieved the most prominent economic gains through the intra-APAC trade.

Our results further demonstrate that the economic and environmental inequity owing to the intraregional trade worsened along time. From 1995 to 2015, the majority of the environmental externalities (i.e., more than 90% of the virtual water flows) were shifted from higher-income to lower-income countries, with a considerable worsening trend for PM_2.5_ (Supplementary Table 3). As for the economic gains associated with the intraregional trade, higher-income countries’ share increased from 38 to 59% from 1995 to 2015. At country level, China experienced the most significant transformation in intra-APAC trade, especially in the virtual trade of water and labor. For virtual blue water flows, China turned from the second largest net exporter of the region in 1995 to the third largest net importer in APAC (5.3 billion m3), largely due to the reverse of the import-export relationship with RoAP. Over the same period, the net virtual water export of India surged by 161%, despite the aggravating water stress concerns. China turned from the largest net supplier of employment (Supplementary Fig. 4) to the second net demander in APAC after two decades, while RoAP showed an opposite trend. For example, the labor- and resource-intensive textile and apparel production industries have been shifted from China to other less-developed countries, such as Vietnam and Bangladesh^50^. Such a role switching stemmed from the rising labor costs in China, which is expected to drive more low-end manufacturers to low-cost foreign economies in the coming years^51,52^. Overall, based on the consistently calculated virtual flows of multiple indicators, we highlight a growing environmental-socio-economic disparity within the APAC region, owing to the intraregional trade. The undesirable environmental externalities are primarily and increasingly shifted from higher to lower-income economies, while higher-income economies achieved more economic gains.

### The role of APAC and intra-APAC trade in globalization

**Fig. 3:**
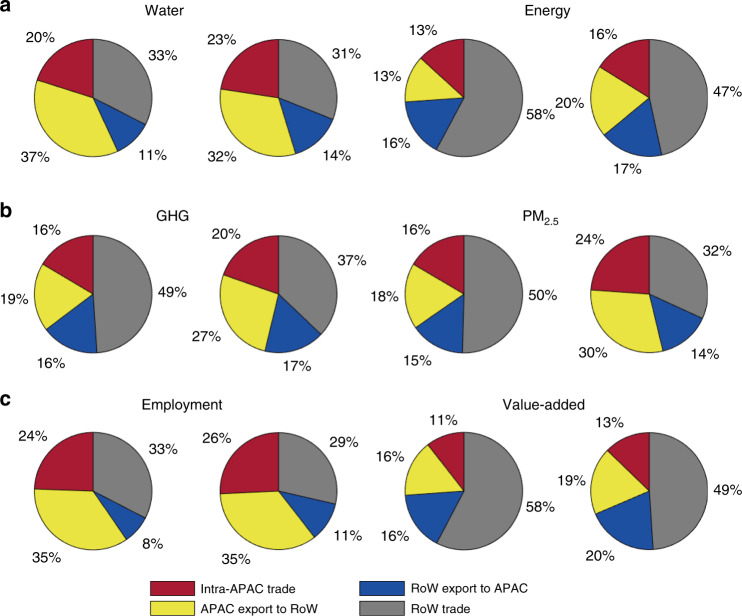
Natural resources, environmental emissions, and socio-economic factors embedded in exports. **a** natural resource, **b** local and global environmental threats, **c** socio-economic effects are shown in three panels. Note, the red and blue sections correspond to the same components in Fig. 1.

APAC is becoming an important player in the globalization process: as natural resource suppliers and manufacturers for the rest of the world, for managing global environmental emissions, and in the labor and monetary markets (Fig. 3). By 2015, APAC-related share of these categories had surpassed 50% (the red, yellow, and blue parts in Fig. 3). In contrast, the international trade without APAC countries (gray in Fig. 3) shrank for all the indicators. Moreover, for all the footprint indicators, the intra-APAC trade and non-APAC’s outsourcing to APAC (red and yellow parts in Fig. 3, respectively) account for a considerable and increasing fraction of the global trade. The former grew from 17 to 20% on average and the latter from 23 to 27% on average. APAC’s outsourcing to non-APAC countries (blue in Fig. 3) only grew slowly, with an average of 14 and 16% in 1995 and 2015, respectively. Earlier studies have proven that the world’s dominating embedded labor flows originate from developing countries, predominately to satisfy the final demand of wealthier economies^23^. Here, we further elucidate that non-APAC economies became more dependent on offshoring the resources, emissions and labor-intensive industries to APAC over the investigation period.

Among the six indicators, we find APAC’s share in global value added was the smallest. In particular, APAC’s economic gains from exports (red + yellow) were significantly smaller (27–32%) than APAC’s resources, emissions, and labor embedded in its exports (40–48%). Intuitively, this may be due to the fact that the APAC region is dominated by population from developing countries, thus resource/labor-intensive and low value-added products dominate the region’s export. In comparison, non-APAC countries are relatively more skilled in producing products and with higher value added and lower intensities of resource and emissions^4^. Also, APAC’s relatively small share in the value-added dimensions of the global supply chains can be a result of the overall low resource efficiency of the region^12^. As resource extractions and environment emissions become increasingly outsourced to the region, where environmental regulations and efficiency measures are only emerging, more environmental impacts will likely be resulted on the global level, offsetting or even reversing the resource efficiency gains and climate change mitigation efforts achieved in developed countries.

On the positive note, from 1995 to 2015, APAC’s resource and environmental intensities declined substantially, both from the perspective of footprint, i.e., footprint per final expenditure, and from the perspective of trade, i.e., direct impacts/gross trade (see Materials and Methods). Such improvement is most noticeable for labor (Table 1). More specifically, over the past two decades, APAC improved faster than the world averages in blue water, PM_2.5_, and labor requirements per expenditure of final consumer, which decreased by 30, 39, and 35%, respectively, while lagging behind the global averages in reduction pace of energy and GHG intensity from footprint perspectives. The intra-APAC trade achieved a reduction in the intensities of energy, GHG and labor by 22, 42, and 34%, respectively. Relatively, this was higher than the world averages over the period. However, the PM_2.5_ embedded in intra-APAC trade per traded values even grew by 7%, whereas the same indicator decreased by 34% at the global scale. This can be explained primarily by the PM_2.5_ trade magnitude in APAC nearly tripling in 2015, much higher than that of the global average (0.8 time). This indicates that the PM_2.5_ issue has become graver for the APAC trade after two decades. The ratio of traded value added to total intratrade values measures the economic gains of trade. Value added is a general indicator of economic performance, yet, it could not evaluate all the advantages of trade, which may cause misleading results in some cases. Therefore, more comprehensive indicators need to be incorporated to assess its advantages.

**Table 1:** Intensity comparison between APAC and the global average from the footprint and trade perspectives.

		Global			APAC			APAC without China		
	Indicator	1995	2015	Change (%)	1995	2015	Change (%)	1995	2015	Change (%)
Footprint	Water	1	0.73	−27%	1.89	1.33	−30%	1.59	1.61	1%
	Energy		0.82	−18%	1.04	1.03	0%	0.80	0.94	17%
	GHG		0.84	−16%	1.21	1.22	2%	0.85	0.98	15%
	PM_2.5_		0.72	−28%	2.01	1.23	−39%	1.00	1.06	6%
	Employment		0.74	−26%	1.84	1.21	−35%	1.23	1.26	2%
Trade	Water	1	0.56	−44%	1.23	0.74	−39%	1.29	0.80	−38%
	Energy		0.81	−19%	0.97	0.76	−22%	0.88	0.94	7%
	GHG		0.59	−41%	1.16	0.67	−42%	0.79	0.69	−12%
	PM_2.5_		0.66	−34%	1.11	1.19	7%	0.64	0.93	47%
	Employment		0.69	−31%	2.03	1.35	−34%	1.62	1.55	−4%
	Value added		0.89	−11%	0.83	0.70	−16%	0.84	0.77	−8%

The 2015 estimates are indexed to the 1995 estimates (1995 estimates = 1). The footprint intensity is calculated as footprints divided by final expenditure (in constant prices). The intensity of global and APAC trade focuses on global trade and intra-APAC trade, respectively, calculated as the direct impacts (e.g., GHG emissions and value added) per gross exports (in constant prices).

Despite APAC’s improvement, APAC’s resource and environmental intensities remain higher in general than the world averages in 2015. The comparison of intensity changes at the national and regional levels was also shown in Supplementary Fig. 5. This can be attributed to the characteristics of the APAC region and may not be altered soon. Specifically, the socio-economic development is supported by resource- and emission-intensive productions in primary industries, especially those linked with capital development and those to satisfy the export demand. The APAC region has a long way to go to achieve a greener and more sustainable trade pathway.

Our result further reveals that, China played a crucial role in APAC’s transformation to greener trade and greener consumption. Generally, without China’s improvement, all the footprint intensities of APAC would exhibit a further increase from 1995–2015, at 8% on average. By contrast, the value is −20% with China in the picture. For the trade intensities, the decline ranges would be much smaller (−1% on average) without China as opposed to with China (−24% on average). Another noteworthy finding is that when eliminating China from APAC, the intensities of footprint and trade in 1995 would be 20–30% lower, implying that China had turned from the lagger to the leader of efficiency improvement in the APAC region.

## Discussion

Despite the strong economic outlook of APAC, both policy makers and researchers remain vigilant over the potential impacts of a shift towards protectionist policies and an increase in geopolitical tensions, as well as the unabated exploitation of natural resources leading to irreversible ecological harm. We explored the coupling features between multiple footprint indicators and affluence of the APAC economies more comprehensively. The APAC economies became increasingly interdependently linked through offshoring more resources, emissions and labor over the investigation period. Moreover, the intraregional trade exacerbated the economic and environmental disparity within the APAC region over the period. With APAC’s increasing involvement in global trade, both the internal and external discrepancies in environmental-social-economic dimensions may become a serious obstacle to APAC’s sustainable development. Improving the efficiency of resource use deserves a central focus of minimizing regional imbalance and promoting sustainable development in the APAC region^53^. Our intensity analysis shows that APAC has made remarkable achievements in the transition to cleaner and greener consumption and trade, but this region still lags behind the global averages in terms of improvement in energy and GHG footprint intensity, as well as the PM_2.5_ intensity of trade. In fact, the consumers and investors in developed regions, such as European countries, have become increasingly conscious in GHG emissions. They ask for more information about products and have implemented various carbon policies, such as carbon tax^54,55^ and cap-and-trade^56^, and are expected to have more policies together with increased consumer preference over products with lower carbon footprint. APAC economies need to further control GHG emissions to maintain and enhance their competitiveness in international trades. In response to those requirements, the consumer-level awareness and behaviour response can be a mitigation option under favourable conditions, but this should be accompanied by concrete initiatives, policies and regulations from industry, commerce and government to eliminate impediments containing structural, economic, and social factors to advance and promote more accessible alternatives^57^.

At present, international climate change mitigation efforts may predominantly depend on resource consumption and emission increases of the industrialized and urbanized countries that are far from the frontier of energy use and with less stringent environmental regulations^58,59^. At the national level, an enhanced integration in improvements in an adjustment of the energy mix, emission control technologies for GHG and air pollution, and changes in the location of production sites are required to reduce emissions across the supply chain. Promotion of resource-efficient production, and innovations of eco-products are vital for mitigating both environmental and resource concerns^60^. At the regional level, alleviating this imbalance requires effective cooperation across the APAC region, i.e., facilitating the policy discussion and co-ordination of policy priorities and strategies. In addition, given some countries’ (i.e., China) notable progress over the research period, advanced techniques can be provided especially for the less-developed APAC countries and regions to alleviate internal imbalance. We argue that failure to make these issues central in policy making in APAC may undermine the region’s competitiveness, the sustainability of intra-APAC trade, and poverty reduction in the long term.

As for future trends, various initiatives (i.e., Asia-Pacific Trade Agreement^61^ and the BRI) will stimulate intraregional and intercontinental trade flows, the economic and environmental effects to the APAC members as they compete for positions in supply chains may become fiercer. The trade of various products such as oil and gas will keep surging to facilitate infrastructure construction, various environmental risks and unforeseen impacts (e.g., overexploitation of natural resources, loss of ecosystem services and biodiversity, etc.) will likely accompany this effort, especially in the poor areas, leading to permanent environmental damages, potential and negative knock-on effects at global scale^60^. To cope with those challenges, more cooperative efforts need to be established to promote a green and sustainable supply chain, and to fulfill the 2030 Agenda for Sustainable Development^62^. Appropriate APAC regional integration could strengthen the cooperation in tackling these common challenges^6^. Given the enormous pressure concerning resource and pollution management for APAC, more research should be conducted in this dynamic region. For example, the supply chain-wide analysis based on MRIO modeling can be applied in recognizing the trade-offs and synergies, understanding how these vital elements are interconnected in the system, and uncovering the critical sectors along the supply chain. Meanwhile, the simultaneous interactions among multiple sectors and international trade should be considered while formulating policies^63–65^.

Our study is subject to three general limitations. First, restricted by the country classification of the IO tables in EXIOBASE 3.6, we treat smaller APAC economies as one aggregated region, RoAP. Thus, details about those economies are missed in our analysis. Second, while we use PM_2.5_ to represent the local pollution impacts, although it captures only part of the impact. The cross-country patterns and trends we charted for PM_2.5_ may look differently for other indicators, such as water eutrophication. Third, our trend analysis for the past two decades was based on two annual snapshots (1995 and 2015), missing the information and insights derivable from the interannual variations.

## Methods

### Footprint calculations based on MRIO analysis

The environmental-social-economic footprints associated with the consumption and trade of the APAC region in 1995 and 2015, respectively, was consistently quantified using EXIOBASE (version 3.6)^66^. EXIOBASE is a MRIO database with a high level of sectoral details, describing the monetary flows and a variety of resource extractions and emissions for 200 product groups and 49 regions and covering the entire global economy. Owing to the limitations of country classifications, we were not able to provide detailed assessments for all countries/regions in the region.

For each footprint indicator *k*, in year *t*, the footprint associated with the final demand of country/region *i* (*Y*_*i,t*_) is calculated using the standard approach of the Leontief inverse:^67^

1 $$E_{k,i,t} = f_{k,t}(I - A_t)^{ - 1}Y_{i,t} + F\_hh_{k,i,t}$$

where *A*_*t*_ is a matrix of direct input coefficients by country/region and product group, *f*_*k*_,_*t*_ is a vector of direct resource or emission coefficients regarding indicator *k*, and *F_hh*_*k,i,t*_ captures households’ direct contributions to *k*. Data of all variables in Eq. 1 were obtained from EXIOBASE. Note that in our approach we did not allocate fixed capital formation to productions. As part of final demand *Y*_*i,t*_, it is considered to contribute to the national footprint of country/region *i*. Although the reality is much more complex: the capital goods produced in year *t* serve human (production) activities for more than one year; the capital goods enable future production activities that contribute to exports and final consumption in countries other than where the capital goods are invested. A few recent research efforts have attempted to better link the emissions embedded in capital goods to human consumption, across time and country^68^. However, the methods are still maturing and are impeded by data availability. All calculations were performed in Matlab and codes are available upon request.

### Footprint indicators system

The six footprint indicators we chose represent different dimensions of sustainability, aiming to provide a more holistic sustainability assessment. More specifically, they cover natural resources use (blue water consumption and energy use), regional and global environmental threats (GHG and PM_2.5_ emissions), and the social and economic effects (employment and value added). GHG includes CO_2_, CH_4_, and N_2_O, based on the 100-year time horizon global warming potential (GWP) according to the fifth assessment report of the Intergovernmental Panel on Climate Change (IPCC)^69^. The energy footprint was based on primary energy use. Employment is measured by the number of people employed.

### Reporting summary

Further information on research design is available in the Nature Research Reporting Summary linked to this article.

## Supplementary information

Supplementary Information

Reporting Summary

## Data Availability

The original data are available from the corresponding author on reasonable request. Source data are provided with this paper. Other datasets generated during this study are available from the corresponding author upon reasonable request.

## Source data

Source Data

## Code availability

The computer codes generating each figure and table in this study are available from the corresponding authors upon reasonable request.
