# Supplementary material for: Environmental-social-economic footprints of consumption and trade in the Asia-Pacific region
Source: Nat Commun. 2020 Sep 8;11:4490. doi: 10.1038/s41467-020-18338-3 (PMC7479111; doi:10.1038/s41467-020-18338-3)
Supplement: Supplementary file 1 — Supplementary Information [file 41467_2020_18338_MOESM1_ESM.pdf]

Supplementary information for

Environmental-Social-Economic Footprints of Consumption and Trade in the Asia-Pacific Region

Yang et al.

Table of contents

Supplementary Methods 3
Supplementary Figures 4
Supplementary Figure 1 4
Supplementary Figure 2 5
Supplementary Figure 3 6
Supplementary Figure 4 7
Supplementary Figure 5 8
Supplementary Tables. 9
Supplementary Table 1 9
Supplementary Table 2 12
Supplementary Table 3 13
Supplementary References 14

Supplementary Methods

Panel data regression. We investigate the relationship between affluence and the four footprint indicators using a fixed effect panel regression model (Eq.1).

$$EP\_pp_{it} = \alpha_i + \beta affluence_{it} + \varepsilon_{it} \quad (1)$$

Where EP_pp_{it} is the per capita footprint of country *i* in the year *t*, $affluence_{it}$ is the average income of country *i* in the year *t*, calculated as per capita GDP adjusted by purchasing power parity (PPP)[1] α_i is the country fixed effect, representing a country's time-invariant characteristics. ε_{it} is the idiosyncratic error term. EP_pp_{it} is calculated using Eq. 1 and EXIOBASE. GDP PPP data are estimated by the World Bank.

We further include the quadratic form of affluence to test the environmental Kuznets Curve (EKC) hypothesis, that environmental pollution first rises and then falls as economic development proceeds, following refs [2,3]

$$EF\_pp_{it} = \alpha_i + \beta_1 affluence_{it} + \beta_2 affluence_{it}^2 + \varepsilon_{it} \quad (2)$$

The turning point of the inverted U curve is calculated as $-\beta_1/2\beta_2$. Results of the regression analysis are shown in Supplementary Table 2, Supplementary Fig. 1 and Supplementary Fig. 2.

Supplementary Figures

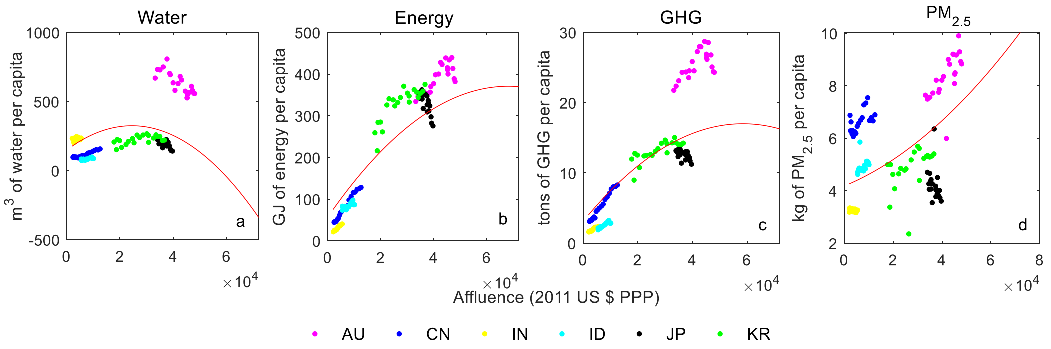

Supplementary Figure 1. Regression plots of per capita footprint associated with the APAC region (six major economies). Panels **a**, **b**, **c**, and **d** correspond to four environmental footprint indicators. Affluence in x-axis equals per capita GDP adjusted by purchasing power parity (PPP) and inflation from the World Bank. AU= Australia, CN=China, IN= India, ID =Indonesia, JP=Japan, KR= South Korea. See the time series data (1995-2015) in source data file.

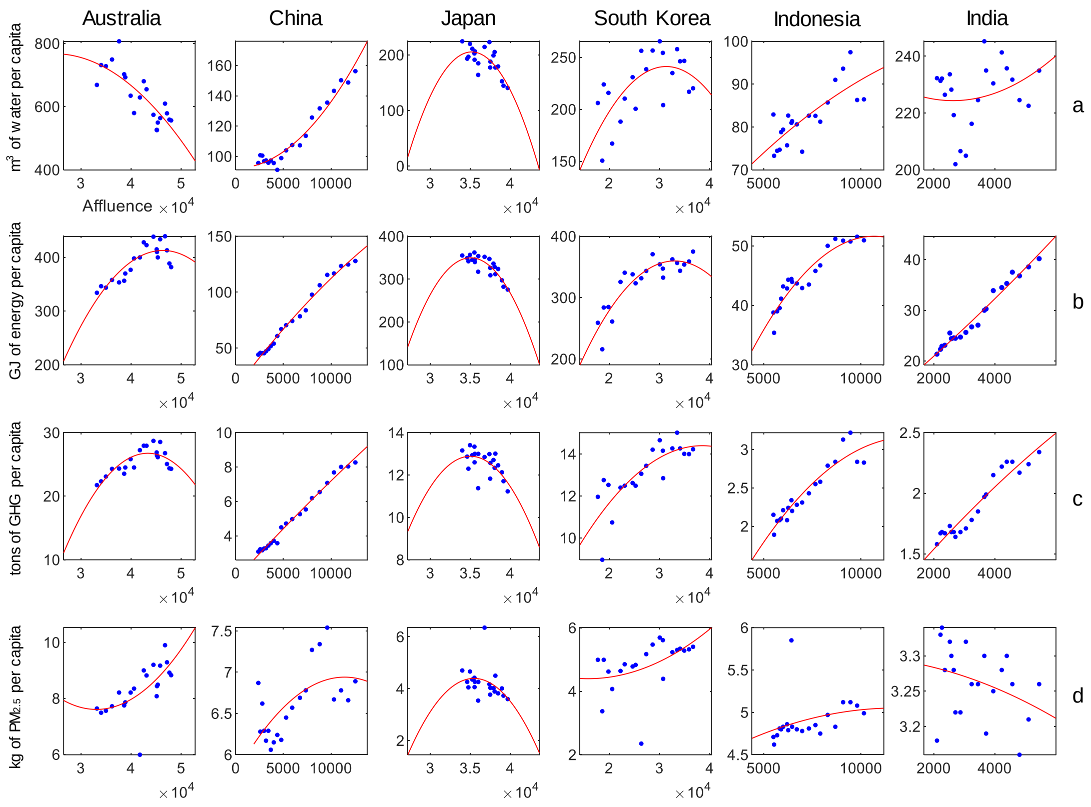

Supplementary Figure 2. Regression plots of per capita footprint associated with individual APAC country. Panels **a**, **b**, **c**, and **d** correspond to four environmental footprint indicators (water, energy, GHG, and $PM_{2.5}$) of individual APAC country. Affluence in x-axis equals per capita GDP adjusted by purchasing power parity (PPP). 2011 is taken as the constant price according to the World Bank. Source data are provided as a Source Data file.

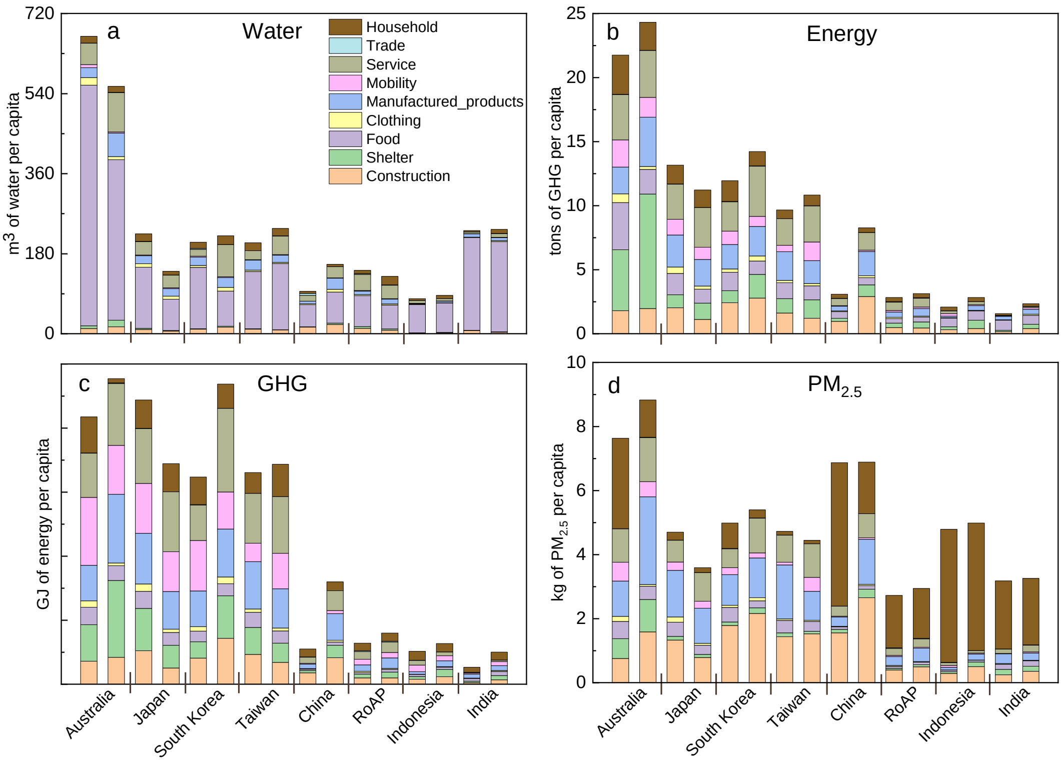

Supplementary Figure 3. Final consumption hotspots in 1995 and 2015. Panels **a**, **b**, **c**, and **d** correspond to four environmental footprint indicators. The bars of two years for the same country/ region are placed adjacently. GHG (including CO_2, N_2O and CH_4) was measured in CO_2 equivalent according to Global Warming Potential. Shelter" refers to the operation and maintenance of residences; "Construction" of buildings is mostly allocated to investments, together with the construction of infrastructure; "Household" refers to the direct emissions that are derived from fuel use by households, which belongs to F_hh[4]. Source data are provided as a Source Data file.

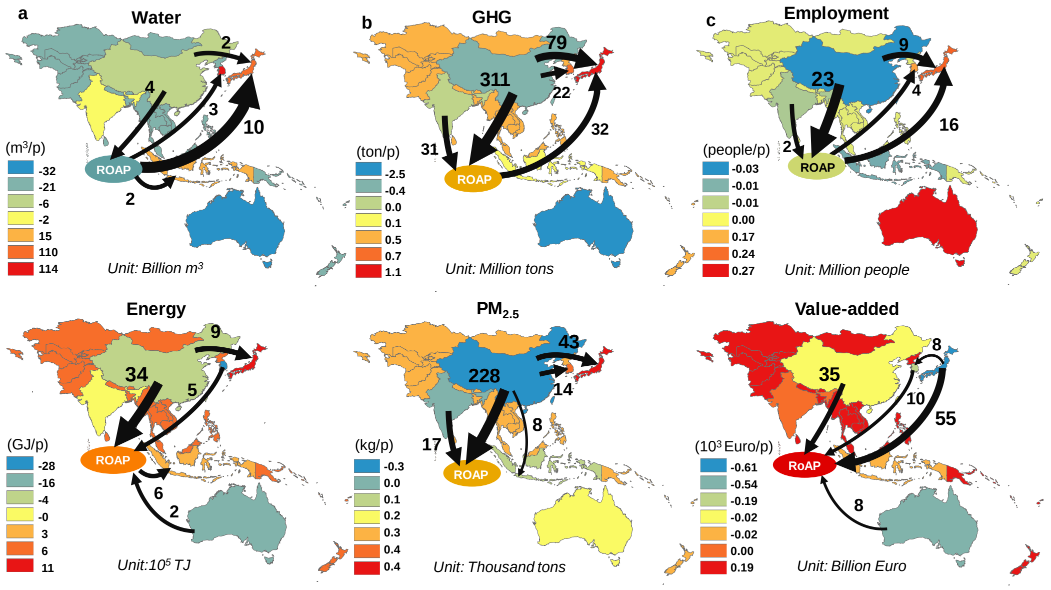

Supplementary Figure 4. Net environmental-social-economic virtual flows of the intra-APAC trade in 1995. The footprint indicators fall into three categories and are presented in **(a)** natural resources, **(b)** local and global environmental threats, and **(c)** socio-economic effects. The width of the arrows in each panel represents the magnitude of the net flow within the APAC region. The background colors indicate the specific net footprint (import-export) per capita of each region/country. The negative net footprint indicates net displacements (of resource use, emissions, labors, and economic value added) to other APAC countries/regions. Valued-added has been adjusted by inflation to make it comparable with Fig.2. Source data are provided in Source Data file.

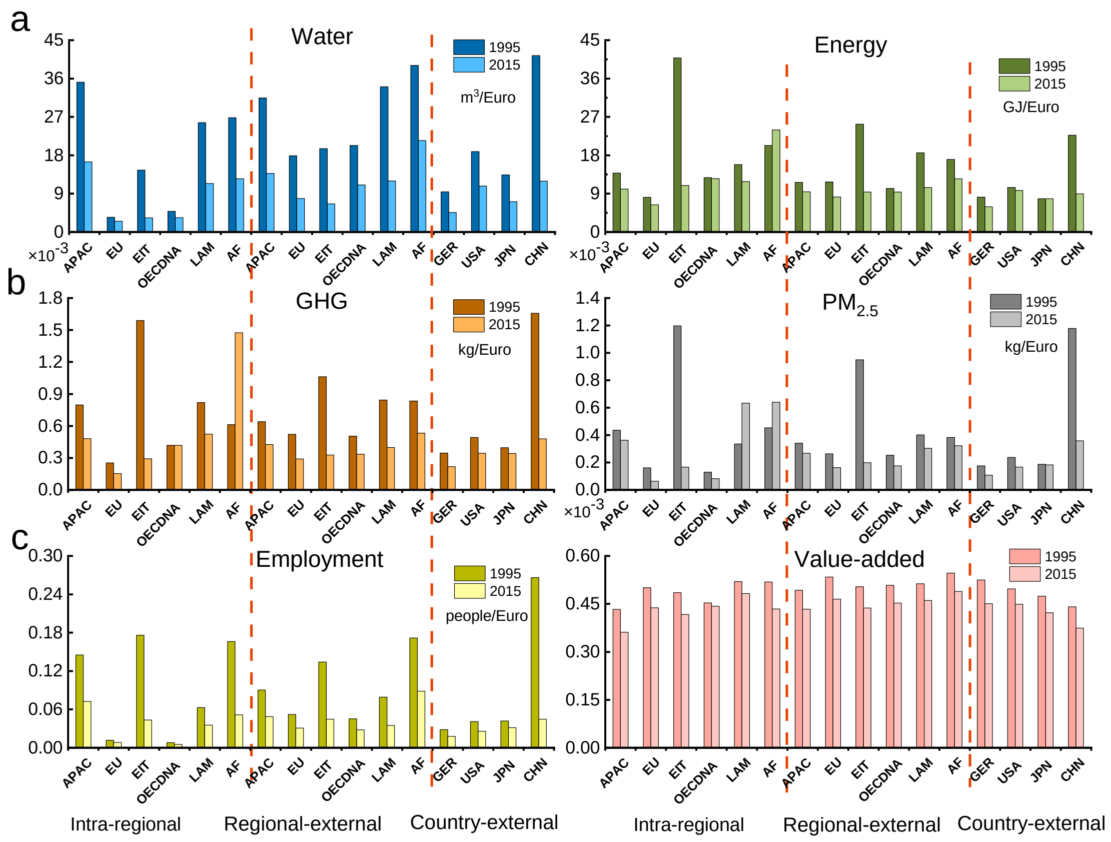

Supplementary Figure 5. Trade intensity per gross output in Euro at country and region scales in 1995 and 2015. The panels **(a)**, **(b)** and **(c)** represent for natural resources, local and global environmental threats, and socio-economic effects, respectively. The intensity calculation considers resource consumed, GHG and $PM_{2.5}$ produced and value-added in bi-directional trade from footprint perspective. For instance, APAC-internal contains bi-directional trade that occurs within the APAC region; APAC-external represents the trade between the APAC region with non-APAC economies; CHN-external represents the trade between China and other economies. EU= The European Union, EIT = The Economy in transition, LAM = Latin America, AF = Africa, OECD = OECD north America. GER= Germany, JPN=Japan, CHN= China. Specifically, EIT contains Bulgaria, Cyprus, Czech Republic, Estonia, Croatia, Hungary, Lithuania, Latvia, Malta, Poland, Romania, Slovenia, Slovakia, and Rest of Europe (RoW Europe). Source data are provided as a Source Data file.

Supplementary Tables

Supplementary Table 1. A brief synthesis of existing footprint studies that touched upon APAC.

NO.	Title of the literature	Footprint indicator	Spatial scope	Key findings related to APAC
1	The employment footprints of nations: uncovering master-servant relationships	Employment	Global	Hong Kong, Singapore, the United Arab Emirates, and Switzerland occupy the top-ranking positions of master countries, whereas many African and Asian countries are servants[5].
2	Drivers of the Growth in Global Greenhouse Gas Emissions	GHG	Global (containing East Asia, India and Indonesia)	15% of all extra GHG emissions between 1995 and 2008 have been emitted in emerging countries (Brazil, Russia, India, Indonesia and China) but were caused by changes in other countries[6].
3	Comparison of bottom-up and top-down approaches to calculating the water footprints of nations	Water	Selected countries (including Australia, China, Japan)	No specific discussion about APAC-related region/countries[7].
4	The emission cost of international sourcing: Using Structural Decomposition Analysis to calculate the contribution of international sourcing to CO2-emission growth	CO_2	Global	The increase in global production-related CO2-emissions has mostly occurred in low-wage countries, in particular China and to a lesser extent India[8].

5	A structural decomposition analysis of global energy footprints	Energy	Selected countries	Most of the global sustainability growth was attributed to Western Europe, Asia and Japan[9].
6	International trade of scarce water	Water	Global	Net exporters are almost exclusively developing, relatively water-scarce countries, however more Middle-Eastern and Central Asian countries rank high after scarcity weighting[10].
7	The role of outsourcing in driving global carbon emissions	CO_2	Global	Carbon leakage is predominantly occurring with trading partners such as China and India, where input restructuring has led to an increase in emissions[11].
8	Trends in global greenhouse gas emissions from 1990 to 2010	GHG	Global (including India, China, Australia, Japan)	Improvements in technology are more than out-run by the combined effect of affluence and population in many countries. Interestingly, this is particularly true for China where affluence has resulted in an almost 8-fold increase in emissions between 1990 and 2010[12].
9	Affluent countries inflict inequitable mortality and economic loss on Asia via $PM_{2.5}$ emissions	$PM_{2.5}$	US, China, Japan, Germany and the UK	Five affluent nations (the US, China, Japan, Germany and the UK) with the highest GDP and import values caused over 1 million people to die prematurely in Asia in 2010 as a result of their induced PM2.5 emissions[13].
10	The "Bad Labor" Footprint: Quantifying the Social Impacts of Globalization	Labor	Global (including seven regions)	Exports from Asia constitute the largest global trade flow measured in the amount bad labor[14].
11	Socioeconomic drivers of global blue water use	Blue water	Global	Demographic changes had considerable accelerating effects on blue water use trends (in particular in the Middle East and North Africa region and in South Asia)[15].
12	International trade linked with disease burden from airborne particulate pollution.	Airborne disease	Global	Most of the world's PM is emitted outside of Asia, but more than half of the global airborne disease burden occurs in just two countries: China and India[16].

13	A structural decomposition analysis of the emissions embodied in trade	CO_2	Forty countries	For the growth of emission embodied in exports, the 7 Asian countries take approximately 53% of the world's total[17].
14	Tele-connecting local consumption to global land use	Land	Global	Based on our analysis, 27–67% of forestland in South-East Asia, China, Russia, Africa and Brazil are displaced for consumption in rich countries[18].
15	Transboundary health impacts of transported global air pollution and international trade	$PM_{2.5}$	Global	30,900 deaths in the 'rest of east Asia' region (which includes Japan and South Korea) were related to emissions in China[19].
16	Measuring the environmental sustainability performance of global supply chains: A multi-regional input-output analysis for carbon, sulphur oxide and water footprints	Carbon, sulphur oxide and water	Global	High water use intensity in China and India in the electricity industry is exacerbated by a national-level ranking of High-Water Stress Risk by the WRI [20].
17	International trade drives global resource use: a structural decomposition analysis of raw material consumption from 1990–2010	Material	Global	Changes in the input structure of the economy tended to increase raw material consumption in many East & South Asian countries (including China, India, and Japan)[21].
18	Trade in occupational safety and health: Tracing the embodied human and economic harm in labour along the global supply chain	Labor	Global	Most African countries and developing countries from southern Asia are the world's top net exporters of accident cases[22].
19	Affluence drives the global displacement of land use	Land	Global	The economies of Europe, Japan, and Korea caused the largest net demand on foreign land mainly to medium-income countries in Latin America, China, and Southeast Asia [23].

Supplementary Table 2. Fixed-effect regression results of panel data

	pp_water	pp_energy	pp_GHG	pp_$PM_{2.5}$	pp_water	pp_energy	pp_GHG	pp_$PM_{2.5}$
affluence	-1.9E-03*	5.48E-03***	2.5E-04***	7.13E-05***	1.44E-02***	9.22E-03***	4.75E-04***	3.83E-05
	(-1.98)	(11.23)	(10.43)	(5.50)	(7.75)	(7.71)	(8.26)	(1.15)
affluence^2					-2.94e-07***	-6.77e-08***	-4.06e-09***	5.98E-10
					(-9.53)	(-3.40)	(-4.24)	(1.08)
	(14.24)	(8.70)	(9.98)	(14.50)	(7.12)	(4.24)	(4.82)	(11.27)
turning point (2011 US$ PPP)					24489.796	68094.530	58497.540	-32023.400
R^2	0.032	0.514	0.478	0.203	0.453	0.558	0.547	0.211
N	126	126	126	126	126	126	126	126

t statistics in parentheses

* $p < 0.1$, ** $p < 0.05$, *** $p < 0.01$

The columns two-five provide the linear regression of the per capita footprint of blue water, energy, GHG and $PM_{2.5}$, and the last four columns are quadratic polynomial regression results of these four footprint indicators by considering the square of affluence. The sample size (N=126) contains data for six major APAC countries: Australia, China, India, Indonesia, Japan, and South Korea from 1995 to 2015.

Supplementary Table 3. The disparity between the rich and poor groups of the APAC region

Indicator	Year	Export of the poor group	Export of the rich group	Net export ratio of the poor group	variation
water（Mm3）	1995	2.32E+04	1.74E+03	86%	1%
	2015	2.16E+04	1.47E+03	87%	
energy（TJ）	1995	4.29E+06	3.42E+06	11%	5%
	2015	8.53E+06	6.13E+06	16%	
GHG (Ton)	1995	2.91E+11	1.35E+11	37%	2%
	2015	4.64E+11	2.07E+11	38%	
$PM_{2.5}$（kg）	1995	1.48E+08	5.71E+07	44%	30%
	2015	3.91E+08	5.81E+07	74%	
employment (1000 people)	1995	5.13E+04	3.60E+03	87%	1%
	2015	6.09E+04	3.84E+03	88%	
value-added (M Euro)	1995	1.16E+05	1.90E+05	rich group's share:38%	21%
	2015	3.28E+05	2.32E+05	rich group's share:59%	

Note: The rich group contains four high-income economies (Australia, Japan, South Korea, and Taiwan), while the less affluent economies, China, India, Indonesia and RoAP are classified into the poorer group. 'export of the rich group' equals the export from rich to poor; 'net export ratio' equals (export of the poor-export of the rich)/(exoprt of the poor + export of the rich).

Supplementary References

1 World Bank, 2016. https://microdata.worldbank.org/index.php/home

2 Dinda, S. Environmental Kuznets curve hypothesis: a survey. *Ecological Economics* **49**, 431-455 (2004).

3 Grossman, G. M. & Krueger, A. B. Economic growth and the environment. *The Quarterly Journal of Economics* **110**, 353-377 (1995).

4 Hertwich, E. G. & Peters, G. P. Carbon footprint of nations: A global, trade-linked analysis. *Environmental Science & Technology* **43**, 6414-6420 (2009).

5 Alsamawi, A., Murray, J. & Lenzen, M. The employment footprints of nations: Uncovering master-servant relationships. *Journal of Industrial Ecology* **18**, 59-70 (2014).

6 Arto, I. & Dietzenbacher, E. Drivers of the growth in global greenhouse gas emissions. *Environmental Science & Technology* **48**, 5388-5394 (2014).

7 Feng, K., Chapagain, A., Suh, S., Pfister, S. & Hubacek, K. Comparison of bottom-up and top-down approaches to calculating the water footprints of nations. *Economic Systems Research* **23**, 371-385 (2011).

8 Hoekstra, R., Michel, B. & Suh, S. The emission cost of international sourcing: using structural decomposition analysis to calculate the contribution of international sourcing to CO2-emission growth. *Economic Systems Research* **28**, 151-167 (2016).

9 Lan, J., Malik, A., Lenzen, M., McBain, D. & Kanemoto, K. A structural decomposition analysis of global energy footprints. *Applied Energy* **163**, 436-451 (2016).

10 Lenzen, M. *et al.* International trade of scarce water. *Ecological Economics* **94**, 78-85 (2013).

11 Malik, A. & Lan, J. The role of outsourcing in driving global carbon emissions. *Economic Systems Research* **28**, 168-182 (2016).

12 Malik, A., Lan, J. & Lenzen, M. Trends in global greenhouse gas emissions from 1990 to 2010. *Environmental Science & Technology* **50**, 4722-4730 (2016).

13 Nansai, K. *et al.* Affluent countries inflict inequitable mortality and economic loss on Asia via PM2. 5 emissions. *Environment International* **134**, 105238 (2020).

14 Simas, M., Golsteijn, L., Huijbregts, M., Wood, R. & Hertwich, E. The "bad labor" footprint: Quantifying the social impacts of globalization. *Sustainability* **6**, 7514-7540 (2014).

15 Soligno, I., Malik, A. & Lenzen, M. Socioeconomic Drivers of Global Blue Water Use. *Water Resources Research* **55**, 5650-5664 (2019).

16 Xiao, Y., Murray, J. & Lenzen, M. International trade linked with disease burden from airborne particulate pollution. *Resources, Conservation and Recycling* **129**, 1-11 (2018).

17 Xu, Y. & Dietzenbacher, E. A structural decomposition analysis of the emissions embodied in trade. *Ecological Economics* **101**, 10-20 (2014).

18 Yu, Y., Feng, K. & Hubacek, K. Tele-connecting local consumption to global land use. *Global Environmental Change* **23**, 1178-1186 (2013).

19 Zhang, Q. *et al.* Transboundary health impacts of transported global air pollution and international trade. *Nature* **543**, 705-709 (2017).

20 Acquaye, A. *et al.* Measuring the environmental sustainability performance of global supply chains: A multi-regional input-output analysis for carbon, sulphur oxide and water

footprints. *Journal of Environmental Management* **187**, 571-585 (2017).
21 Plank, B., Eisenmenger, N., Schaffartzik, A. & Wiedenhofer, D. International trade drives global resource use: A structural decomposition analysis of raw material consumption from 1990–2010. *Environmental Science & Technology* **52**, 4190-4198 (2018).
22 Alsamawi, A., Murray, J., Lenzen, M. & Reyes, R. C. Trade in occupational safety and health: Tracing the embodied human and economic harm in labour along the global supply chain. *Journal of Cleaner Production* **147**, 187-196 (2017).
23 Weinzettel, J., Hertwich, E. G., Peters, G. P., Steen-Olsen, K. & Galli, A. Affluence drives the global displacement of land use. *Global Environmental Change* **23**, 433-438 (2013).
