# Supplementary material for: Environmental-social-economic footprints of consumption and trade in the Asia-Pacific region
Source: Nat Commun. 2020 Sep 8;11:4490. doi: 10.1038/s41467-020-18338-3 (PMC7479111; doi:10.1038/s41467-020-18338-3)
Supplement: Supplementary file 2 — Reporting Summary [file 41467_2020_18338_MOESM2_ESM.pdf]

nature research

Corresponding author(s): Yutao Wang

Last updated by author(s): Jul 25, 2020

Reporting Summary

Nature Research wishes to improve the reproducibility of the work that we publish. This form provides structure for consistency and transparency in reporting. For further information on Nature Research policies, see our Editorial Policies and the Editorial Policy Checklist.

Statistics

For all statistical analyses, confirm that the following items are present in the figure legend, table legend, main text, or Methods section.

n/a	Confirmed	
x		The exact sample size (n) for each experimental group/condition, given as a discrete number and unit of measurement
x		A statement on whether measurements were taken from distinct samples or whether the same sample was measured repeatedly
x		The statistical test(s) used AND whether they are one- or two-sided<br>*Only common tests should be described solely by name; describe more complex techniques in the Methods section.*
x		A description of all covariates tested
x		A description of any assumptions or corrections, such as tests of normality and adjustment for multiple comparisons
x		A full description of the statistical parameters including central tendency (e.g. means) or other basic estimates (e.g. regression coefficient) AND variation (e.g. standard deviation) or associated estimates of uncertainty (e.g. confidence intervals)
x		For null hypothesis testing, the test statistic (e.g. F, t, r) with confidence intervals, effect sizes, degrees of freedom and P value noted<br>*Give P values as exact values whenever suitable.*
x		For Bayesian analysis, information on the choice of priors and Markov chain Monte Carlo settings
x		For hierarchical and complex designs, identification of the appropriate level for tests and full reporting of outcomes
x		Estimates of effect sizes (e.g. Cohen's d, Pearson's r), indicating how they were calculated

Our web collection on statistics for biologists contains articles on many of the points above.

Software and code

Policy information about availability of computer code

Data collection	MATLAB R2019a
Data analysis	MATLAB R2019a

For manuscripts utilizing custom algorithms or software that are central to the research but not yet described in published literature, software must be made available to editors and reviewers. We strongly encourage code deposition in a community repository (e.g. GitHub). See the Nature Research guidelines for submitting code & software for further information.

Data

Policy information about availability of data

All manuscripts must include a data availability statement. This statement should provide the following information, where applicable:

- Accession codes, unique identifiers, or web links for publicly available datasets
- A list of figures that have associated raw data
- A description of any restrictions on data availability

The original data are available from the corresponding author on reasonable request. All the source data underlying Figs. 1–3, Supplementary Figs. 1–5 are provided as a Source Data file. Other datasets generated during this study are available from the corresponding author upon reasonable request.

Field-specific reporting

Behavioural & social sciences study design

All studies must disclose on these points even when the disclosure is negative.

Study description	This study is the first to analyse the environmental and socioeconomic implications of the growth in welfare and trade in Asia-Pacific (APAC). It utilizes quantitative methods with multi-regional input-output tables.
Research sample	The global multi-regional input-output (MRIO) table applied in this study is a database with a high level of sectoral details, describing the monetary flows and a variety of resource extractions and emissions for 200 product groups and 49 regions and covering the entire global economy.
Sampling strategy	The footprint indicators in this study representing sustainability covers environmental-social-economic dimensions
Data collection	The MRIO table used in this study were the latest EXIOBASE 3.6, which were available upon reasonable request from the corresponding authors.
Timing	The MRIO table used in this study is for the year 1995-2015.
Data exclusions	No data was excluded from the analysis.
Non-participation	No participant declined.
Randomization	Participants were not allocated into experiment groups.

Reporting for specific materials, systems and methods

We require information from authors about some types of materials, experimental systems and methods used in many studies. Here, indicate whether each material, system or method listed is relevant to your study. If you are not sure if a list item applies to your research, read the appropriate section before selecting a response.

Materials & experimental systems

n/a	Involved in the study	
☒	☐	Antibodies
☒	☐	Eukaryotic cell lines
☒	☐	Palaeontology and archaeology
☒	☐	Animals and other organisms
☒	☐	Human research participants
☒	☐	Clinical data
☒	☐	Dual use research of concern

Methods

n/a	Involved in the study	
☒	☐	ChIP-seq
☒	☐	Flow cytometry
☒	☐	MRI-based neuroimaging
